# Feasibility of Implementing Cancer-Specific Community-Based Exercise Programming: A Multi-Centre Randomized Trial

**DOI:** 10.3390/cancers14112737

**Published:** 2022-05-31

**Authors:** Margaret L. McNeely, Kirsten Suderman, Janice L. Yurick, Kathryn Nishimura, Christopher Sellar, Paula A. Ospina, Edith Pituskin, Harold Lau, Jacob C. Easaw, Matthew B. Parliament, Anil A. Joy, S. Nicole Culos-Reed

**Affiliations:** 1 Department of Physical Therapy, University of Alberta, Edmonton, AB T6G 2G4, Canada; kirsten.suderman@ualberta.ca (K.S.); knishimu@ualberta.ca (K.N.); csellar@ualberta.ca (C.S.); pospina@ualberta.ca (P.A.O.); 2 Cancer Care Alberta, Alberta Health Services, Edmonton, AB T5J 3E4, Canada; matthew.parliament@albertahealthservices.ca; 3 Department of Oncology, Faculty of Medicine, University of Alberta, Edmonton, AB T6G 2R3, Canada; pituskin@ualberta.ca (E.P.); jay.easaw@albertahealthservices.ca (J.C.E.); anil.joy@albertahealthservices.ca (A.A.J.); 4 Cross Cancer Institute, Alberta Health Services, Edmonton, AB T6G 1Z2, Canada; yuricks@telus.net; 5 Faculty of Nursing, University of Alberta, Edmonton, AB T6C 1C9, Canada; 6 Tom Baker Cancer Centre, Alberta Health Services, Calgary, AB T2N 4N2, Canada; harold.lau2@albertahealthservices.ca; 7 Cumming School of Medicine, University of Calgary, Calgary, AB T2N 4N1, Canada; nculosre@ucalgary.ca; 8 Faculty of Kinesiology, University of Calgary, Calgary, AB T2N 1N4, Canada

**Keywords:** cancer, exercise, implementation, randomized controlled trial

## Abstract

**Simple Summary:**

Exercise has emerged as an effective therapeutic modality for improv-ing the health, quality of life and overall survival of individuals with cancer. We assessed the feasibility of implementing a cancer-specific community-based exercise program in advance of a planned large-scale exercise implementation study. Findings supported high interest in, and benefits for fitness outcomes among participants both during and immediately following com-pletion of cancer treatment. Lessons learned from this feasibility trial included the need for clos-er attention to implementation processes, and adaptations to physical fitness and outcome measures to better fit the community setting.

**Abstract:**

Background: There is growing recognition of the importance of reporting preliminary work on the feasibility of a trial. The present study aimed to assess the feasibility of (1) a proposed fitness testing battery, and (2) processes related to the implementation of cancer-specific exercise programming in a community setting. Methods/Design: A randomized controlled implementation feasibility trial was performed in advance of a large-scale implementation study. Eligible participants within 18 months of a cancer diagnosis were randomized to immediate or delayed community-based exercise at YMCA locations in Calgary and Edmonton, Canada for an 8-week period. The primary outcome for the trial was the feasibility of the physical fitness testing battery, defined as a 70% or greater completion rate across the 24-week study period. The Reach, Effectiveness, Adoption, Implementation and Maintenance (RE-AIM) framework was used to evaluate processes related to implementation of the exercise program across the two sites. Results: Eighty participants were recruited, 73 (91%) completed the 8-week trial, and 68 (85%) completed the 16- and 24-week follow-ups. Sixty participants (75%) completed the full physical fitness test battery at each time point, and 59 (74%) completed the patient-reported outcome measures. Statistically significant between-group differences were found in favor of the exercise group for functional aerobic capacity, upper and lower extremity strength, and symptoms. Differences were found between the sites, however, in completion rates and processes related to program implementation. Discussion: Findings suggest the need for minor adaptations to the physical fitness battery and outcome measures to better fit the community context. While findings support feasibility, context-specific challenges related to implementation processes were identified.

## 1. Introduction

Exercise has emerged as an effective therapeutic modality for improving health outcomes, as well as the quality and quantity of life of individuals with cancer [1,2]. Specifically, research evidence supports the beneficial effects for physical functioning, body composition, symptoms, and disease outcomes [3,4,5,6]. Recent consensus documents on exercise for individuals with cancer are available to guide the healthcare professional on prescriptive programs by cancer type, treatment, symptoms, and outcomes [2,7,8]. The translation of this evidence into standard cancer care, however, remains elusive [9]. Moreover, the lack of evidence on the effectiveness, affordability, and accessibility of exercise programs, which serve as important benchmarks to inform healthcare resource allocation decisions, present challenges in attaining sustainable funding for implementation [10].

As survivorship numbers grow, implementing cancer-specific exercise programs in community-based settings offers a potentially low-cost, accessible solution to support large-scale exercise rehabilitation and behavior change [11,12]. While early evidence on community-based exercise programs shows promise [13,14,15,16,17], a key area of contention in the exercise oncology field revolves around the effectiveness and sustainability of cancer-specific community-based programs [18]. Criticisms of the focus on community-based initiatives include a lack of concordance with patients’ preferences for home-based interventions, as well as poor attendance and high dropout rates reported from community programs [18,19,20,21]. Thus, further study is needed to provide contextually relevant and timely information on factors that can enhance or impede implementation success [22,23]. Assessing the feasibility of an implementation strategy offers the opportunity to evaluate both the outcomes and processes involved and refine the intervention and its delivery format prior to program spread and scale [22,24].

Our interdisciplinary team conducted a feasibility implementation trial in preparation for our large-scale Alberta Cancer Exercise (ACE) hybrid effectiveness-implementation study [25]. Qualitative findings related to participant engagement and our integrated knowledge translation approach have been previously published [26]. Here, we share the ACE feasibility trial results and our experience with initial community-based program implementation. 

### Objectives

The objectives of this feasibility implementation trial were to: (1) inform the utility (i.e., practical implementation feasibility) and acceptability of the physical fitness battery; (2) identify potential refinements to program reach and intervention delivery; and (3) explore preliminary program effectiveness at the level of the participant [27].

## 2. Materials and Methods

A randomized controlled feasibility trial design was utilized to support evaluation of program effectiveness on fitness and patient-reported outcomes. Approval was received from the Health Research Ethics Board of Alberta: Cancer Committee and the University of Calgary Health Research Ethics Board. Informed consent was obtained from all individual participants included in the trial. The trial was registered at clinicaltrials.gov on 5 January 2015 (NCT02330575).

### 2.1. Recruitment

From March 2015 to August 2016, recruitment took place in two centers in Alberta, Canada—at the Cross Cancer Institute/University of Alberta (Edmonton) and Tom Baker Cancer Centre/ University of Calgary (Calgary). The exercise intervention was delivered at YMCA community fitness centers in Edmonton (Don Wheaton Family YMCA) and Calgary (Saddletowne YMCA). Specific clinics at the sites (e.g., breast cancer, head and neck cancer) and education classes (e.g., fatigue management) were targeted for participant recruitment. A research coordinator was available, on site, to facilitate the referral process. 

### 2.2. Eligibility

For the purposes of the feasibility trial, individuals (≥18 years of age) within 18 months of a cancer diagnosis were recruited. Participants could be receiving or have completed cancer treatment (e.g., surgery, chemotherapy, or radiation therapy). Oncologist approval was required prior to enrollment. Potential participants completed cancer-specific screening and the Physical Activity Readiness Questionnaires (PAR-Q+) to determine appropriateness for the community-based exercise program [28].

Exclusion criteria included:

(1) Presence of metastatic disease.
(2) Any uncontrolled or serious comorbid conditions that would preclude participation in exercise testing or training.
(3) Women who were pregnant.
(4) Greater than 18 months from cancer diagnosis.

Participants were stratified by site (Calgary or Edmonton) and cancer category (i.e., breast, head and neck, neurological or other type of cancer) and randomized to early (intervention group) or delayed community-based exercise programming (standard care group). Participants randomized to the immediate exercise group took part in the ACE supervised program at the respective YMCA location in their city for an 8-week period. Following the 8-week supervised intervention period, participants had the option to continue on a ‘fee for service’ basis at the YMCA site. Participants allocated to the standard care group served as the ‘control group’ for a 16-week period. After completion of the 16-week follow-up testing, participants in this group were offered participation in the 8-week ACE supervised program. For both groups, a final follow-up assessment was conducted at 24 weeks. Participant evaluations took place on trial entry (T0), at the end of week 8 (T1), week 16 (T2), and week 24 (T3) (Supplementary Materials Figure S1). 

### 2.3. Interventions

#### 2.3.1. Group 1: Standard Care: Physical Activity Counseling

Participants randomized to the standard care group received information on the importance of exercise during and following cancer treatment, and how to incorporate physical activity into their day-to-day life. Participants were encouraged to progressively increase their physical activity with the goal of reaching the levels specified in public health guidelines (i.e., at least 150 min of moderate intensity aerobic exercise each week) [7].

#### 2.3.2. Group 2: Supervised Community-based Exercise 

Participants randomized to the immediate exercise group took part in a combination of aerobic, resistance, balance, and flexibility exercises twice weekly for an 8-week period. The participant exercise sessions were conducted in small groups of 5 to 10 participants. Two options for community-based exercise programming existed: group fitness classes or supervised fitness center access. Participants took part in a combination of aerobic, resistance, balance, and flexibility exercises delivered in a standardized circuit-type class setting or group personal training format, twice weekly for a minimum of 60 min per session (approximately 3–4 metabolic equivalent (MET) units per session) for an 8-week period. The program included options for low-to-moderate intensity exercise set at 3–4 MET units per session (360–480 MET-minutes per week) and was progressed in intensity to 4–5 METs over the 8-week program duration (480–600 MET-minutes per week) [29]. In terms of intensity, this would be similar to prescribing walking at a comfortable pace (4 km per hour) initially and then slowly progressing to brisk walking (6 km per hour) over an 8-week period [25]. Participants were encouraged to perform one additional self-directed exercise session per week at the fitness facility or at home as a means to achieving public health guidelines for physical activity (i.e., 500–1000 MET-minutes per week or at least 150 min of moderate intensity aerobic exercise each week) [25,29]. The program was administered by qualified exercise specialists at the YMCA sites in Calgary and Edmonton. The respective YMCA exercise specialists completed a cancer-specific 16-h in-person training course involving content related to cancer biology, cancer incidence, treatment and treatment-related effects, exercise evidence and prescription for cancer survivors, and health behavior change. An experienced cancer-trained clinical exercise physiologist or physical therapist was available to provide support to the respective exercise specialist. 

### 2.4. Primary Outcome: Feasibility of Fitness Testing Battery

The primary outcome for the trial was feasibility, defined as a 70% completion rate of the required physical fitness testing battery across each time point over the 24-week trial [30]. Our goal was to evaluate if the fitness testing components were feasible for delivery in a community-based setting. 

### 2.5. Secondary Outcomes: Recruitment and Completion Rate, Program Reach and Effectiveness 

Recruitment was determined by the participation rate, defined as the number of participants agreeing to participate divided by the number of eligible participants contacting the research team (target of 30% or higher). Where possible, reasons for nonparticipation were collected. We recorded the number of dropouts and withdrawals, and if provided, reasons for non-completion of tests and for leaving the trial (target of 75% completion). Attendance to the exercise sessions was tracked as a marker of acceptability (target of 75% or higher) [31]. 

### 2.6. Reach (Individual Level)—Collected Prospectively, Evaluated at Trial Completion

The RE-AIM components of Reach, Effectiveness and Implementation at the *level of the individual* were evaluated as potential outcome indicators for the ACE program, including:

1. Methods used to recruit participants and referral sources.
2. Inclusion and exclusion criteria for the program: evaluation of numbers excluded and reasons. Demographic and medical characteristics were collected, and the Physical Activity Stage of Change Questionnaire was administered to evaluate the participant’s status in terms of attitudes and behaviors towards increasing physical activity.
3. Program costs associated with trial oversight, research coordinator presence in clinics and education classes, and costs for exercise testing and programming.

### 2.7. Effectiveness (Individual Level)—Collected at Baseline, Week 8, Week 16, and Week 24

1. Intervention outcomes: Health-related aspects of both physical fitness and quality of life in individuals with cancer were assessed by having participants in the ACE program complete the comprehensive fitness battery as per the standards of the Canadian Society of Exercise Physiology [32] as well as patient-reported outcomes both before (baseline) and after the exercise program (at weeks 8, 16, and 24). Measure of functional aerobic capacity: YMCA submaximal cycle test OR Balke submaximal treadmill test OR the six-minute walk test [33] (choice in test was determined based on participant preference).
  - Musculoskeletal fitness: grip strength [34,35], eight repetition-maximum for bench press and leg press [36], and sit-and-reach (flexibility) [32].
  - Cancer-related symptoms: Memorial Symptom Assessment Scale [37].
  - Quality of Life: Functional Assessment of Cancer Therapy-General scale [38].
2. Safety was monitored during exercise testing and training by the supervising qualified exercise specialist, who was responsible for recording any serious adverse events. Participants were asked to self-report any issues, injuries, or falls both related and unrelated to exercise participation to the research coordinator at the respective site.

### 2.8. Allocation Concealment and Method of Randomization 

A staff member from the Rehabilitation Research Centre at the University of Alberta, independent of the trial research team, generated the randomization sequence for each site and stratified group. The sequence was concealed from all trial personnel. The random group assignments were placed in numbered, sealed opaque envelopes. Participants were randomized on a 1:1 ratio to the intervention group or standard care by the respective research coordinator after completion of the baseline fitness testing. 

### 2.9. Protection from Sources of Bias

At each measurement point starting at the baseline assessment and including the 8-week, 16-week, and 24-week follow-ups, Independent Assessors unaware of treatment allocation (blinded) performed the objective physical fitness measurements. Trial coordinators administered the symptom assessment and quality of life questionnaires. Blinding of participants and exercise specialists was not possible. Trial participants were free to withdraw from the study at any time. Participants remained in their randomized group to preserve the intent-to-treat principle.

### 2.10. Sample Size

A sample size of 80 allowed for an estimated completion rate of 70% to within a 95% confidence interval of +/− 15%. Our estimated sample size for the planned larger-scale implementation study was 800; therefore, a sample size of 80 (~10%) was deemed appropriate to evaluate feasibility [39,40]. 

### 2.11. Analysis Plan

Descriptive analyses were performed for participant demographic and exercise related variables, as well as program costs. Feasibility outcomes were compared using Pearson’s Chi-square tests for categorical data. Point estimates and confidence intervals were determined for physical fitness, symptom, and quality of life outcomes based on complete case analyses at 8- and 16-week follow-ups. All outliers were retained as reflecting variability inherent in the cancer population during this phase in the cancer treatment trajectory. To explore differences between groups post-intervention (i.e., at 8 weeks) and at follow-up (i.e., at 16 weeks), an analysis of covariance (ANCOVA) was used with baseline measures and time since diagnosis as covariates [41]. Effect sizes and 95% confidence intervals were calculated using the adjusted mean difference between groups at the end of the intervention. Hedges’ g was used to describe the magnitude of the change with 0.2 representing a small effect, 0.5 a moderate effect, and 0.8 a large effect [42]. Data were analyzed using SPSS version 26. 

## 3. Results

**Figure 1:**
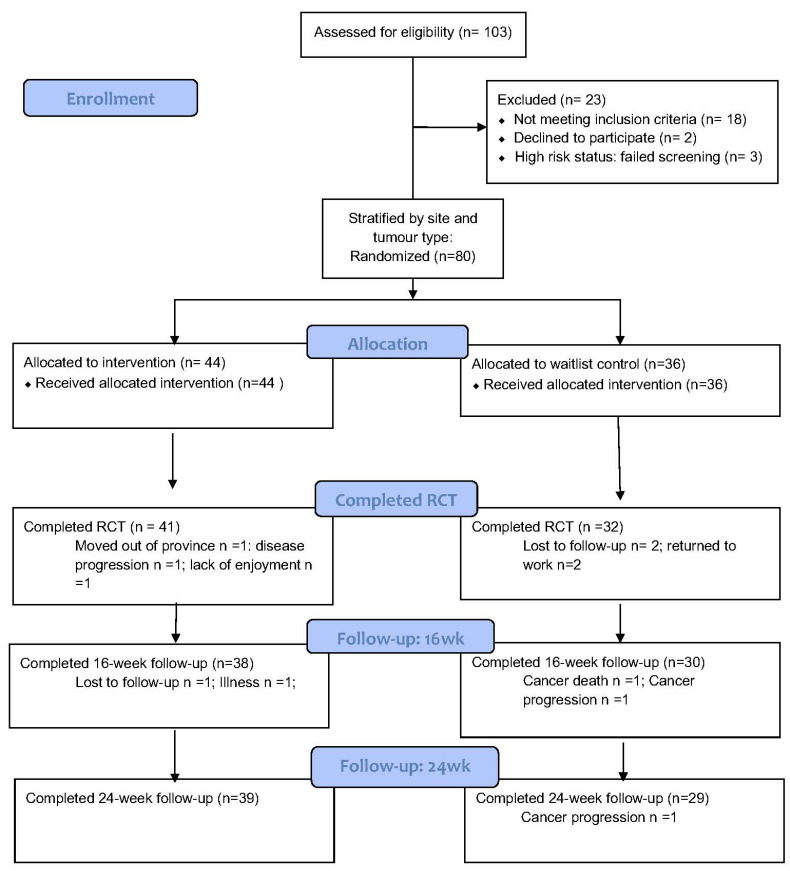
Study Flow.

The study took place between March 2015 and November 2016. A total of 103 individuals with cancer were screened and 82 were deemed eligible for the trial (Figure 1). Of the 82 eligible participants, 80 (98%) were recruited, 73 (91%) completed the trial, and 68 (85%) completed the 16- and 24-week follow-ups. Details on participant characteristics are displayed in Table 1.

### 3.1. Utility and Acceptability of the Physical Fitness Battery

Sixty participants (75%) had full data for all physical fitness battery components at each time point (Table 2). For the submaximal test of functional aerobic capacity, 43 (54%) opted to complete the six-minute walk test, 16 (20%) the YMCA submaximal cycle ergometer test, 13 (16%) the Balke submaximal treadmill test, and 8 (10%) did not complete any test of functional aerobic capacity (breast cancer *n* = 2, head and neck cancer *n* = 6). Baseline to 24-week completion rates for the required fitness battery components were 80% for functional aerobic capacity, 84% for grip strength, 81% for sit-and-reach, and 81% for body composition. No statistically significant differences were found between groups in the proportion of participants completing the full physical fitness test battery over the 24-week trial period (*p* = 0.299); however, statistically significant differences were found between the sites, with 56% and 89% of participants completing the full battery for Calgary and Edmonton sites, respectively (*p* = 0.001). Fifty-nine participants (74%) completed the patient-rated outcomes at each time point (Supplementary Materials Table S2). 

Twenty-two participants (50%) randomized to immediate exercise opted to continue on a fee-for-service basis from weeks 9–24. Twenty-nine control participants (81%) opted to cross-over to exercise after the 16-week follow-up, and 26 (72%) completed the 8-week cross-over intervention. (Further details are provided in the Supplementary Materials Tables S1 and S2). 

### 3.2. Program Reach

Fifty percent of the participants enrolled in the trial were diagnosed with breast cancer, and 71% were female. A majority of patients self-referred to the program (68%) were between 13- and 18-months post-diagnosis (56%), and in the post-cancer treatment phase (74%). Of patients referred, the most common reason for ineligibility was being >18 months from diagnosis (*n* = 18; 90%). Further data on program reach and costs are described in Table 3. 

### 3.3. Effectiveness 

**Figure 2:**
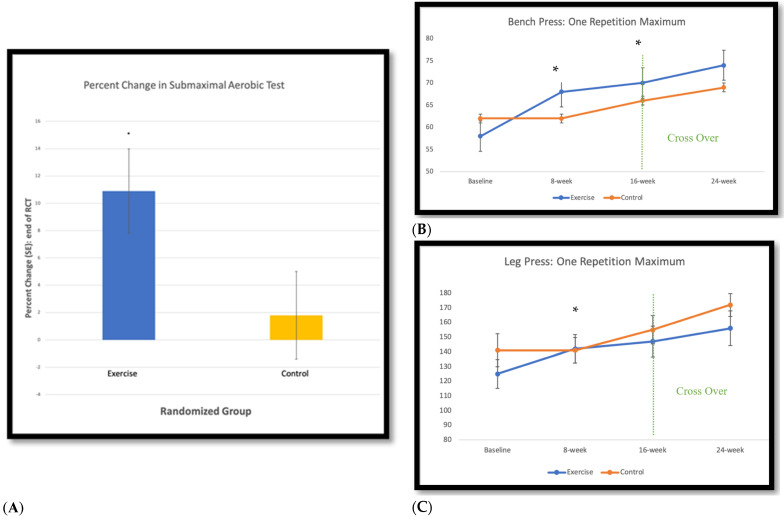
Findings for Functional Aerobic Capacity and Upper and Lower Extremity Strength Tests. (**A**) Percent change T0-T1: Functional Aerobic Capacity, * between group difference: *p* < 0.05; (**B**) Upper Extremity: Bench Press, * between group difference: *p* < 0.05; (**C**) Lower Extremity: Leg Press, * between group difference: *p* < 0.05.

No statistically significant differences were found between groups for the respective individual tests of functional aerobic capacity; however, a significant between-group difference was found in favor of the exercise group for percentage change in aerobic capacity from baseline to 8 weeks (Figure 2A). Statistically significant between-group differences were also found in favor of the exercise group for the change in 1RM leg press at 8 weeks, and the 1RM bench press at 8 and 16 weeks (Figure 2B,C). No statistically significant differences were found between groups for other fitness, body composition, or self-reported outcomes, with the exception of significantly fewer reported symptoms on the Memorial Symptom Assessment Scale following the 8-week intervention in favor of the exercise group (Table 4). 

### 3.4. Attendance and Adverse Events

No serious adverse events occurred at either location. Minor adverse events related to the exercise program included: shoulder pain (*n* = 2), foot pain (*n* = 1), and back pain (*n* = 2). No participants withdrew as a result of their musculoskeletal pain, and all issues were resolved with rest and modification of exercises. Exercise participants reported a mean of 115 min per week of moderate intensity exercise during the intervention period. Attendance at exercise sessions for the Edmonton site was 81%. Primary reasons for non-attendance included: (1) participant concerns over potential exposure to bacteria and viruses in a public fitness facility when on cancer treatment; (2) return to work schedules and ongoing medical appointments conflicting with ability to attend group exercise sessions; (3) accessibility issues due to the downtown locations of the community fitness centers, including parking, construction in the area, traffic, and seasonal winter road conditions. 

## 4. Discussion

### 4.1. Feasibility 

This feasibility trial was performed to guide planning for a subsequent large-scale community-based implementation study [25]. A primary finding was the high completion rate for the physical fitness battery in the community-based setting supporting feasibility. While overall findings support feasibility, statistically significant differences were found in completion rates between the sites. This was an unanticipated finding that was likely due to context-specific differences in implementation processes. In Edmonton, the research team’s delivery of the community-based program was more consistent with an explanatory research approach [23]. Research staff were actively involved in the program, including through oversight of data collection, monitoring of participant adherence, and provision of onsite support to the community exercise specialist delivering the intervention, features consistent with prior YMCA implementation approaches [43,44]. Benefits of this approach included documentation of program adaptations, collection of adherence data, as well as reasons for non-adherence and participant withdrawals. In contrast, research staff in Calgary focused their efforts on clinic recruitment to programming, and, as such, a higher percentage of participants at this site reported oncologist referral to the trial. Participant adherence and program adaptations were less formally collected—features more consistent with practical implementation [45]. The primary benefits of this pragmatic approach included lower costs for onsite programming and data collection that is likely more reflective of real-world settings [45]. Taken together, the findings demonstrated the need for standardization of recruitment and referral processes as well as on-site support for data collection in the community setting for the subsequent larger-scale implementation study [46]. Given the challenges of implementing exercise into routine cancer care, close examination of effectiveness and implementation processes were deemed equal priorities to support future spread and scale [24,47,48].

In terms of acceptability of the fitness test battery, the majority of participants in the trial opted to complete the six-minute walk test over a submaximal treadmill or bike test. The six-minute walk test was found to be simple to administer, practical for the community setting, and could be done efficiently in small groups of participants at the same time. Although the treadmill and cycle ergometer tests offered opportunities to tailor aerobic exercise prescriptions [20], these tests required two assessors and almost double the time to administer, increasing the time burden and costs associated with testing. Thus, the six-minute walk test was deemed acceptable to participants, and most appropriate for future implementation. Outcomes such as grip strength, body composition, and sit-and-reach tests were feasible to perform and time efficient; however, similar to findings of prior research, no statistically significant improvements were found from exercise over standard care [20]. The lack of significance seen in these and other outcomes, however, may be due to the heterogeneity among cancer types and treatment status, as well as the relatively short intervention period. 

### 4.2. Program Reach and Intervention Delivery

As commonly seen in exercise oncology studies, the vast majority of participants in this trial were diagnosed with breast cancer, in the early stages following cancer treatment completion, and in the contemplation or preparation stage of exercise behavior change. Despite the growing evidence for the benefit of exercise, overall physical activity levels of individuals with cancer both during and following cancer treatment remain low [49]. Therefore, improving uptake among those less inclined to exercise will require the integration of strategies such as oncologist recommendation [18], healthcare provider counseling on exercise options [18,26,50], and a tailored multicomponent approach to address exercise-related barriers [51,52]. Additionally, extending participant inclusion into a longer recovery time period after cancer treatment would open opportunities for participants dealing with ongoing cancer-related impairments. A variety of approaches to physical activity and exercise promotion are likely needed that may involve, for example, hospital and clinic-based services for individuals requiring greater supervision, and independent and virtual home-based exercise options that are more accessible to a wider clinical population [50,53].

### 4.3. Preliminary Effectiveness 

Prior group-based and supervised settings have been reported to show superior benefits for functional aerobic capacity, muscular fitness, and patient-reported outcomes [54,55]. Our findings support high completion and medium-to-large positive effects for outcomes of functional aerobic capacity, upper and lower body muscular strength, and cancer symptoms. Thus, this study adds to the growing body of evidence supporting the benefit of supervised community-based programs for key physical fitness outcomes [17,44,56,57,58,59]. Moreover, community-based programs offer the opportunity to bring individuals with cancer together to create their own supportive environment and can help build social networks that encourage positive behavior change [60]. Fifty percent of exercise participants in this trial opted to continue on a fee-for-service basis, with identified facilitators including the support and mentorship of cancer peers, as well as having trained and qualified exercise professionals leading and supporting the classes [26].

Findings of a recent meta-analysis of cancer-specific exercise programs suggest small overall effects across outcomes, with medium-to-large effects found when programs were supervised and involved contact time above 24 h across program implementation (i.e., 12 weeks with 2 sessions per week) [55]. Thus, a longer intervention period may be needed to realize benefits across a broader range of outcomes [61]. When examining our program costs, we noticed that by simply reducing the number of follow-up testing time points from 4 to 3 assessments, funds could be redirected to extend the community-based exercise program from 8 to 12 weeks. 

### 4.4. Strengths and Limitations 

This study has several strengths and limitations. A strength of the study was the randomized controlled trial design that allowed for evaluation of the relative benefit of exercise over standard care, at a time point in the cancer treatment trajectory when individuals with cancer may be naturally recovering from the effects of cancer treatments [62]. The retention rate of participants completing the randomized controlled trial (91%) and follow-up testing (85%) are much higher than those reported in prior publications of cancer-specific community-based exercise programs [20,21,56,63,64]. Findings suggest, however, the need for minor adaptations of the physical fitness battery and outcome measures to better fit the community context, as well as closer oversight of data collection to inform effectiveness. Trial-related limitations include the relatively short intervention period and the heterogenous group of individuals with cancer in terms of cancer types and treatment status. Limits to external generalizability include a predominantly Caucasian, female, and highly educated sample. Notwithstanding these limitations, this trial provides valuable insight into the challenges of implementing cancer-specific community-based exercise programs. 

## 5. Conclusions

Overall, this trial produced the following conclusions: (1) closer attention is needed to site-specific contextual factors to inform program adaptations and understand factors affecting program adherence and completion; (2) expanding eligibility to encompass a longer recovery period post-cancer treatment will extend program reach; (3) in-clinic counseling strategies are needed to support systematic referral; and (4) increased exercise program contact time may facilitate beneficial effects across a broader range of physical, functional, and patient-reported outcomes. Moving forward, effectiveness-based research is needed to inform cancer-specific, community-based implementation intervention design and performance.

## Figures and Tables

**Table 1:** Baseline Demographics.

**Variable**	**Category**	**Exercise (*n* = 44)**	**Control (*n* = 36)**
		**Mean (SD)/No. (%)**	**Mean (SD) or No. (%)**
Age	Age on study entry	56.0 (11.9)	56.3 (10.6)
Type of cancer	Breast	22 (50)	18 (50)
	Head and Neck	11 (25)	13 (36)
	Neurological	5 (11)	2 (6)
	Lymphoma	2 (5)	-
	Prostate	-	1 (3)
	Gastrointestinal	2 (5)	2 (6)
	Lung	2 (5)	-
Cancer treatment status	On active treatment	11 (25)	9 (25)
	Completed treatment	31 (70)	27 (75)
	Not reported	2 (5)	-
Gender No. (%)	Female	32 (73)	25 (69)
	Male	12 (27)	11 (31)
Marital Status	Married/Common Law	32 (73)	25 (69)
	Divorced/Separated	4 (9)	8 (22)
	Single/Widowed	8 (18)	3 (8)
Education	High School	7 (16)	10 (28)
	University	33 (75)	23 (64)
	Graduate School	4 (9)	3 (8)
Ethnicity	Caucasian	34 (77)	28 (78)
	Asian	6 (14)	3 (8)
	African	-	2 (6)
	Arab	1 (2)	-
	First Nations	-	2 (6)
	Not reported	3 (7)	1 (3)
Location of residence	Edmonton/Calgary city limits	34 (77)	29 (81)
	Within 50 km from Edmonton/Calgary	8 (18)	5 (14)
	Not reported	2 (5)	2 (6)
Body Mass Index Category	Normal weight	19 (43)	18 (50)
	Overweight	12 (27)	9 (25)
	Obese	13 (30)	9 (25)
Physical Activity Stage of Change	Contemplation	15 (34)	14 (39)
	Preparation	17 (39)	17 (47)
	Action	10 (23)	3 (8)
	Not reported	2 (5)	2 (6)

**Table 2:** Completion rates of individual components of the physical fitness battery.

Outcome	Overall	Edmonton	Calgary
Functional aerobic capacity			
Baseline	72 (90%)	46 (100%)	26 (88%)
8-week	70 (88%)	46 (100%)	24 (71%)
16-week	64 (80%)	43 (93%)	21 (62%)
24-week	64 (80%)	43 (93%)	21 (62%)
Grip Strength			
Baseline	79 (99%)	46 (100%)	33 (97%)
8-week	71 (89%)	46 (100%)	26 (76%)
16-week	66 (83%)	43 (93%)	21 (62%)
24-week	67 (84%)	43 (93%)	21 (62%)
Flexibility (sit and reach)			
Baseline	79 (99%)	46 (100%)	32 (94%)
8-week	73 (89%)	45 (98%)	28 (82%)
16-week	65 (81%)	41 (89%)	24 (71%)
24-week	65 (81%)	41 (89%)	24 (71%)
Body Composition			
Baseline	80 (100%)	46 (100%)	34 (100%)
8-week	71 (89%)	46 (100%)	25 (74%)
16-week	62 (78%)	41 (89%)	21 (62%)
24-week	65 (81%)	43 (93%)	22 (65%)
Completion of all Components of the Fitness Battery *	60 (75%)	41 (89%)	19 (56%)

* Overall Completion rate: Exercise vs. Control groups: *p* = 0.299; Edmonton vs Calgary: *p* = 0.001.

**Table 3:** Evaluation of Processes: Program Reach.

Reach Component	Implementation Determinant
Referral sources	Edmonton: Oncologist referral (*n* = 3); Self-referral *(n* = 43) Calgary: Oncologist referral (*n* = 19); Self-referral (*n* = 11); Not reported *(n* = 4)
Numbers excluded and reasons	22% exclusion rate Primary reason for exclusion: >18 months post diagnosis
Characteristics of study participants	Highly educated 78% Caucasian 71% female 50% with breast cancer diagnosis 59% in preparation or action stage of change *
Program Costs	Training of Community Exercise Specialists: $1500 Study coordination and recruitment: 4–6 h per week: $22,500 per site = $45,000 Program fees: $800/8-week session × 10 programs = $8000 ($100 per participant) Outcome assessment: $35 per session × 4 timepoints = $10,200 ($140 per participant)
Per participant cost	$790 Canadian: includes costs associated with recruitment, program oversight, participant screening, program coordination and registration, fitness testing, and delivery of the 8-week exercise intervention

* Stage of change: preparation stage = making small changes in exercise behavior; action stage = exercising for less than 6 months.

**Table 4:** Secondary Outcomes.

	T0 Baseline	T1: Post-Intervention	Adjusted between-Group Mean Difference: T0 to T1	T2: 16-Week Follow-Up	Adjusted between-Group Mean Difference: T0-T2	Estimated Effect Size Baseline to Post-Intervention
Outcome	Mean (SD)	Mean (SD)	Exercise versus Control Mean Change [95% CI]	Mean (SD)	Exercise versus Control Mean Change [95% CI]	Hedges’ g (95% CI); Interpretation
Grip Strength combined (lbs) Control (*n* = 31) Exercise (*n* = 40)	129.1 (39) 132.7 (41)	131.7 (40) 137.4 (53)	2.6 [−4.3, 9.6]	135.7 (42) 135.4 (43)	−3.7 [−10.7, 3.3]	0.17 [−0.31, 0.64] Small effect
Sit and Reach (cm) Control (*n* = 33) Exercise (*n* = 40)	20.8 (10) 19.7 (11)	22.7 (12) 22.0 (10)	1.8 [−1.6, 5.2]	20.9 (13) 22.0 (10)	2.0 [−1.4, 5.4]	0.27 [−0.2, 0.74] Small effect
Body Mass Index Control (*n* = 31) Exercise (*n* = 40)	25.8 (5.9) 27.2 (6.0)	26.6 (5.6) 26.2 (7.3)	−0.9 [−2.4, 0.7]	26.9 (5.7) 26.0 (7.4)	−1.2 [−3.5, 1.1]	0.23 [−0.24, 0.7] Small effect
FACT-G (score: 0–108) Control (*n* = 32) Exercise (*n* = 40)	77.8 (14) 79.6 (13)	78.8 (15) 83.1 (13)	3.3 [−1.6, 8.2]	79.9 (16) 82.7 (12)	2.6 [−3.3, 8.4]	0.25 [−0.22, 0.73] Small effect
MSAS symptoms (no.) Control (*n* = 35) Exercise (*n* = 40)	9.9 (7) 10.5 (6)	10.5 (7) 9.0 (6)	−2.0 [−4.1, −0.1] *	9.2 (7) 8.4 (5)	−1.0 [−3.3, 1.3]	−0.49 [−0.95, −0.03] Medium effect
MSAS Total Score (0–4) Control (*n* = 32) Exercise (*n* = 38)	0.5 (0.4) 0.7 (0.4)	0.6 (0.3) 0.5 (0.4)	−0.1 [−0.5, 0.3]	0.79 (0.8) 0.71 (0.7)	−0.16 [−0.5, 0.2]	−0.46 [−0.95, 0.03] Medium Effect

Adjusted for baseline score and time from diagnosis. Functional Assessment of Cancer Therapy: General scale (FACT-G): higher scores = better quality of life. Memorial Symptom Assessment Scale (MSAS): higher scores = more symptoms/higher symptom profile; number (no.); * p < 0.05.

## Data Availability

Data will be accessible via the University of Alberta Cancer Rehabilitation Clinic Dataverse at: https://dataverse.scholarsportal.info/privateurl.xhtml?token=5bc8a006-3c48-4d1b-a6cc-cf6a5b253e42 accessed on 14 April 2022.

## Supplementary Materials

The following supporting information can be downloaded at: https://www.mdpi.com/article/10.3390/cancers14112737/s1, Figure S1: Trial Schema; Table S1: Study Completion Rates by Follow-up Time Point by Tumour Type, Group, and Site; Table S2: Completion Patient-rated outcomes and optional tests.

## Abbreviations

ACE: Alberta Cancer Exercise; YMCA: Young Men’s Christian Association.
