# Supplementary material for: Feasibility of Implementing Cancer-Specific Community-Based Exercise Programming: A Multi-Centre Randomized Trial
Source: Cancers (Basel). 2022 May 31;14(11):2737. doi: 10.3390/cancers14112737 (PMC9179478; doi:10.3390/cancers14112737)
Supplement: Supplementary file 1 [file cancers-14-02737-s001.zip › cancers-1707412-supplementary.pdf]

Supplementary Material

Figure S1. Trial Schema

Group/ Weeks	Weeks 1-8	Weeks 9-16	Weeks 16-24
Exercise	ACE Program Supervised 2x per week; no cost	Fee for service/ Self-directed exercise	Fee for service/ Self-directed exercise
Standard Care	Counseling on physical activity	→	ACE Program: Supervised 2x per week; no cost

T0----------------------------T1-----------------------------T2---------------------------T3

RCT **Follow-up Period** **Cross-over**

Table S1. Study Completion Rates by Follow-up Time Point by Tumour Type, Group, and Site

Site	Tumor Type	Group Allocation	Baseline	8 weeks End of RCT	16 weeks	24 weeks Cross-over
**Edmonton (n =46)**	Breast (n =25)	Exercise	13 (100%)	13 (100%)	13 (100%)	12 (92%)
		Control	12 (100%)	11 (92%)	10 (83%)	11(92%)
	HNC (n =10)	Exercise	4 (100%)	4 (100%)	4 (100%)	4 (100%)
		Control	6 (100%)	6 (100%)	6 (100%)	5 (71%)
	Neurological (n =2)	Exercise	2 (100%)	2 (100%)	2 (100%)	2 (100%)
		Control	--------	--------	-------	-------
	Other (n =9)	Exercise	6 (100%)	6 (100%)	6 (100%)	6 (100%)
		Control	3 (100%)	3 (100%)	3 (100%)	3 (100%)
	**Subtotal by site**		46 (100%)	45 (98%)	44 (96%)	43 (93%)
**Calgary (n =34)**	Breast (n =15)	Exercise	9 (100%)	8 (89%)	7 (78%)	7 (78%)
		Control	6 (100%)	6 (100%)	6 (100%)	5 (71%)
	HNC (n =14)	Exercise	7 (100%)	7 (100%)	5 (71%)	7 (100%)
		Control	7 (100%)	4 (57%)	4 (57%)	4 (57%)
	Neurological (n =5)	Exercise	3 (100%)	1 (33%)	1 (33%)	1 (33%)
		Control	2 (100%)	2 (100%)	1 (50%)	1 (50%)
	**Subtotal by site**		34 (100%)	28 (82%)	24 (71%)	25 (74%)
	**Overall**		**80 (100%)**	**73 (91%)**	**68 (85%)**	**68 (85%)**

Table S2. Completion Patient-rated outcomes and optional tests

Outcome	Overall	Edmonton	Calgary
All patient-rated outcome measures			
Baseline	70 (88%)	46 (100%)	26 (76%)
8-week	67 (84%)	46 (100%)	25 (74%)
16-week	62 (78%)	43 (93%)	24 (71%)
24-week	59 (74%)	41 (89%)	18 (53%)
Upper extremity 8 RM testing (optional)			
Baseline	72 (90%)	45 (98%)	27 (79%)
8-week	72 (90%)	45 (98%)	27 (79%)
16-week	70 (88%)	43 (93%)	27 (79%)
24-week	69 (86%)	41 (89%)	18 (53%)
Lower extremity 8 RM testing (optional)			
Baseline	63 (79%)	40 (87%)	23 (68%)
8-week	63 (79%)	40 (87%)	23 (68%)
16-week	55 (69%)	36 (78%)	19 (56%)
24-week	57 (71%)	36 (78%)	21 (62%)
